# Suit for Malpractice

**Published:** 1846-10

**Authors:** 


*Suit for Malpractice.*—The Boston Med. and Surg. Journal says, that "Mr. Whitney had a leg amputated by Dr. Cowles, of Marcellus, N. Y., in Sept. 1844, in such a manner, he contended in a suit before the court recently in session at Syracuse, that he had suffered much pain and loss of time ever since—and therefore he asked for damages. The jury after being out 4 hours returned a verdict in his favor of $500!" The editor significantly adds, "what surgeon in New-York State, after this and some previous decisions dare perform an operation?"

We learn that Dr. Cowles will probably appeal from this verdict to a higher tribunal. We are promised the notes of this trial at some future time.

